# Alternative Splicing Role in New Therapies of Spinal Muscular Atrophy

**DOI:** 10.3390/genes12091346

**Published:** 2021-08-28

**Authors:** Jan Lejman, Grzegorz Zieliński, Piotr Gawda, Monika Lejman

**Affiliations:** 1 Student Scientific Society, Laboratory of Genetic Diagnostics, Medical University of Lublin, 20-093 Lublin, Poland; lejmjan@gmail.com; 2 Department of Sports Medicine, Medical University of Lublin, 20-093 Lublin, Poland; grzegorz.zielinski@umlub.pl (G.Z.); piotr.gawda@umlub.pl (P.G.); 3 Laboratory of Genetic Diagnostics, Medical University of Lublin, 20-093 Lublin, Poland

**Keywords:** alternative splicing, therapies, Spinal Muscular Atrophy

## Abstract

It has been estimated that 80% of the pre-mRNA undergoes alternative splicing, which exponentially increases the flow of biological information in cellular processes and can be an attractive therapeutic target. It is a crucial mechanism to increase genetic diversity. Disturbed alternative splicing is observed in many disorders, including neuromuscular diseases and carcinomas. Spinal Muscular Atrophy (SMA) is an autosomal recessive neurodegenerative disease. Homozygous deletion in 5q13 (the region coding for the motor neuron survival gene (*SMN1*)) is responsible for 95% of SMA cases. The nearly identical *SMN2* gene does not compensate for SMN loss caused by *SMN1* gene mutation due to different splicing of exon 7. A pathologically low level of survival motor neuron protein (SMN) causes degeneration of the anterior horn cells in the spinal cord with associated destruction of α-motor cells and manifested by muscle weakness and loss. Understanding the regulation of the *SMN2* pre-mRNA splicing process has allowed for innovative treatment and the introduction of new medicines for SMA. After describing the concept of splicing modulation, this review will cover the progress achieved in this field, by highlighting the breakthrough accomplished recently for the treatment of SMA using the mechanism of alternative splicing.

## 1. Introduction

Splicing is an essential part of pre-mRNA maturation in a eukaryotic cell. That process consists of excising noncoding intronic sequences from the initial product of gene transcription and ligating remaining exons before translation to protein [1]. Splicing reaction is controlled by the spliceosome, the macromolecular ribonucleoprotein complex. Determination of the beginning and end of the intron, which is marked by the 5′ and 3′ splice sites (5′ss and 3′ss), plays a key role in the splicing mechanism. Specific sequences are recognized by the spliceosome, the macromolecular ribonucleoprotein structure that catalyses splicing [2]. An alternatively spliced gene is a source of multiple mRNA isoforms, which increases coding potential of the eukaryotic genome. Alternative splicing is regulated by the cis-acting splicing regulatory elements (SREs) that recruit trans-acting factors in a sequence-unique manner [3]. Trans-acting RNA binding proteins (RBPs) bound to an intronic or exonic splice enhancer (ISE or ESE) stabilize spliceosome formation and lead to exon recognition and retention. Analogically RBPs bound to intronic or exonic splice silencing motifs (ISS or ESS) preclude the formation of the spliceosome and promote exon removing [4]. The most common RBPs are proteins rich in serine/arginine (SR) rests and the heterogeneous ribonucleoprotein (hRNP). Due to tissue-specific RBPs bounding, final products from the same gene primary transcript can be different depending on the tissue. the tissue-specific RBPs bounding, final products from the same gene primary transcript can be different depending on the tissue. The binding of these proteins is a variable process resulting in diverse combinations of included or excluded introns and exons [5]. SR proteins are responsible for phosphorylation, which regulates their localization and activity [6]. Oxidative stress affects mutations within splicing regulatory sequences or disturbed expression of splice factors, which causes a growing number of diseases and has an emerging role in aging [7,8,9]. Loss of balance in the splicing process influences the development of neurodegenerative diseases, renitis pigmentosa, Prader-Willi syndrome, familial adenomatous polyposis, breast or lung cancer [10,11,12,13,14,15,16]. The aim of this review is to present the novel forms of therapies of spinal muscular atrophy (SMA) based on alternative splicing regulation mechanisms.

## 2. Spinal Muscular Atrophy (SMA)

SMA is a congenital neurodegenerative disorder with an autosomal recessive inheritance, characterized by loss of motor neurons leading to progressive muscle weakness [17]. Knowledge about SMA has changed considerably since the first reports of patients with this disease, written by Werdnig (1891) [18] and Hofmann (1893) [19]. The SMA incidence is about 1 in 6000 to 11,000, with a carrier frequency of *SMN1* mutations from 2 to 3% (1 in 40) in the general population [20,21,22]. In Cuba, a six-year study was conducted to investigate the prevalence of type I SMA in people of different ethnicities. The results of the study suggest that type I SMA is less common in the African American group [23]. According to the statistics of the Polish SMA Foundation, one in 35 inhabitants of Poland carry the *SMN1* gene mutation, and the disease phenotype will appear on average in every 7000 children born in Poland [24]. Based on the progression and variability of symptoms, SMA was divided into five types, from congenital lethal (SMA0) to adult onset (SMA4) [25]. The clinical phenotype of SMA is heterogeneous, ranging from severe to mild. It is generally divided into three main subtypes: Type I (also called Werdnig Hoffmann disease), Type II, and Type III (also called Kugelberg Welander disease). However, these phenotypes are viewed more as a continuum rather than as separate subtypes, and further subtypes are sometimes observed at both ends of the spectrum. Type 0 SMA is a very severe form with onset in utero, limited, or missing movements, contractures, and a requirement for assisted mechanical ventilation at birth and death before six months of age, while Type IV SMA is a mild late (adult) form that has a normal life span [20,21].

In most cases this disease develops due to mutations in the gene *SMN1* (survival of motor neuron 1), SMN T, telomeric, located on chromosome 5q13.2 [26]. The majority of the patients (92–95%) have a homozygous deletion of *SMN1* [20,21]. The intragenic mutations within *SMN1* are responsible for the remaining 5% of cases [27]. In some severe cases of SMA, loss of the *NAIP* (neuronal apoptosis protein inhibitor), *GTF2H2A* (general transcription factor IIH, p44), and *SERF1A* (small EDRK-rich factor 1A, *H4F5A*) genes are also observed [28,29,30,31,32,33]. A study by Ahn, Eun Ji et al. on a group of 33 Korean patients suggests that coexisting deletions of *SMN1* and *NAIP* are connected with earlier onset of symptoms and poor prognosis in SMA patients [34]. The transcription of *SMN1* produces a functionally complete mRNA that encodes SMN protein. Significantly fewer SMN proteins come from the *SMN2* gene. Only 10–15% of total *SMN2* transcripts are full-length mRNA [35]. Thus, *SMN2* is identical to *SMN1,* except for a single C-T substitution in exon 7. This substitution promotes 80 to 85% splicing during transcription and consequent exon 7 deletion [35]. The *SMN2* genes are not functionally equivalent. The ability of the *SMN2* gene to modify the course of the disease is regulated by epigenetic factors that, through DNA methylation, have the ability to silence the gene. In patients with different types of SMA, differences in methylation levels are observed at positions −296 and −290 in the island 2 CpG of *SMN2*. A milder disease course correlates with lower methylation levels [36]. It is worth noting that truncated mRNA causes similarly truncated non-functional proteins. Patients with SMA lack *SMN1* and therefore they depend on the residual *SMN2* production of a functional SMN protein for α function of the motor neuron and subsequent survival [21]. The SMN protein is localized in all eukaryotic cells and has been shown to have a pivotal role in homeostatic cellular pathways in all cells [37]. According to hypotheses, the SMN protein in the cytoplasm was shown to have an important role in the transport of mRNA through axons and transport of containing b-actin ribonucleoprotein complexes. Another hypothesis states that the SMN protein takes part in synthesis of small nuclear RNA (snRNA) and therefore plays a key role in the formation of a spliceosome that removes introns from pre-mRNA into functional mRNA [38,39]. As motor neurons are sensitive to malfunctioning of the spliceosome directly or indirectly through misspliced mRNAs, any damage to motor neurons results in the development of dysfunctions in proteins essential for neuronal function [21].

## 3. Mechanisms of *SMN2* Splicing Regulation Targeted by Therapeutics

In human cells, there are two nearly identical genes responsible for SMN protein production. The presence of two *SMN* genes is attributed to large tandem chromosomal duplication [40]. In the region of this duplication, on the long arm of chromosome 5 (5q13.2) lie four protein-encoding genes: *SMN, NAIP, GTF2H2A,* and *SERF1A*. The duplicated genes are identical to their partner gene (*SERF1B*), differ in a low number of nucleotides (*SMN2*) or are pseudogenes (*ΨGTF2H2B* and *ΨNAIPΔ5*) [41]. Both SMN genes consist of 10 exons (1,2A,2B,3–6,6B,7,8). It is worth noting that Exon 6b is a new discovery and is generated by exonification of the Alu element in intron 6 [42]. Under certain conditions such as starvation, hypoxia, or oxidative stress, transcription of these genes may proceed differently [8,43]. Factors that regulate SMN levels and modify transcription are tissue-specific [44].

**Figure 1:**
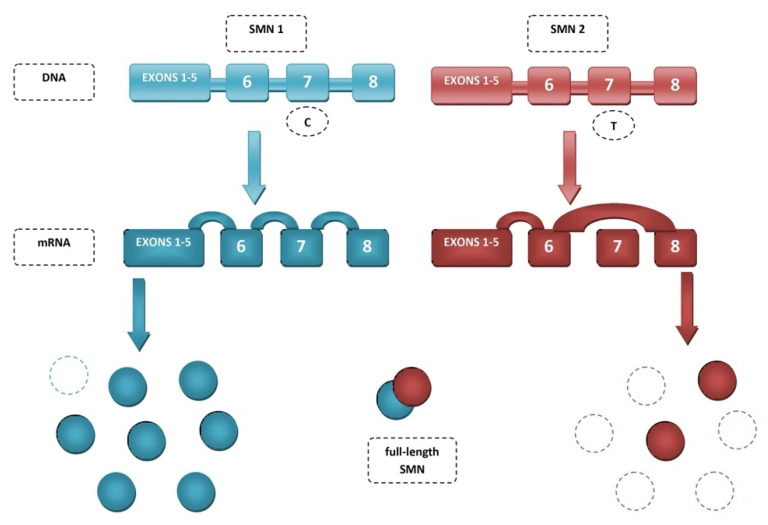
Splicing of *SMN* genes.

The key difference between these genes lies in the splicing of exon 7. The amino acids encoded in exon 7 are responsible for SMN stability as they determine the crucial C-terminus of the protein. In the *SMN2* gene due to alternative splicing, exon 7 is more often skipped, resulting in more of the truncated, partially functional and unstable SMNΔ7 protein than full-length SMN [45,46]. The primary reason exon 7 is excluded is C-to-T substitution at position 6 of exon 7 (C6U) (Figure 1). Mutation or deletion of the *SMN1* gene is a major cause of spinal muscular atrophy, through deficiency of SMN [39]. Restoring exon 7 inclusion has therapeutic benefits proven in mouse models [47].

**Figure 2:**
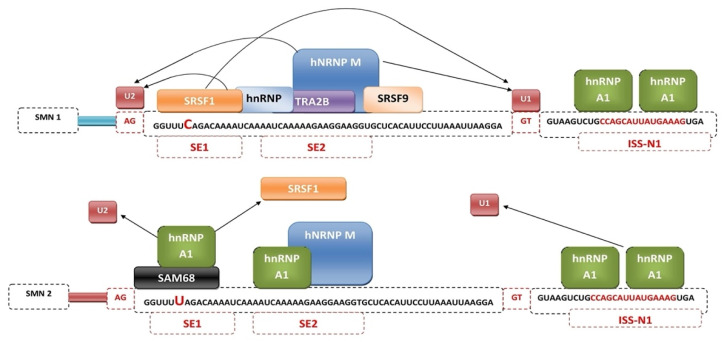
Mechanism of regulation of *SMN1* and *SMN2* gene splicing by splicing factors.

Mechanisms regulating exon 7 splicing are good potential therapeutic targets. The best described to date splicing factors, binding directly to exon 7 splicing enhancer regions SE1 and SE2 are serine/arginine-rich splicing factor 1 (SRSF1) and transformer 2 protein homolog β (Tra2B) [48]. The best-known negative regulators of exon 7 splicing are heterogeneous nuclear ribonucleoprotein A1 (hnRNP A1) [49] and src-associated substrate in mitosis 68 (Sam68) [50]. C6U substitution results in hnRNA A1 or Sam68 being bound to SE1 in place of the positive regulator. Exon 7 exclusion can also occur through binding of hnRNP A1 to SE2 sequences or the intronic silencer sequence N1 (ISS-N1) [51] (Figure 2). Other factors showing altering splicing activity include SRp30c [52], TDP-43 [53], TIA1 [54], hnRNP Q [55], hnRNP G [56].

Exon 7 skipping may be caused by increased activity of a regulatory sequence located at the 3′ end of exon 7, called terminal stem loop 2 (TSL2). It exhibits inhibitory activity, probably by competing with U1 snRNP for a binding site [57]. The inhibitory effect of TSL2 was confirmed by observing the effect of U40G or A54C substitution on exon 7 splicing. Separately, they disrupted TSL2 by promoting exon 7 incorporation, but combined they reproduced the structure of TSL2 and thus inhibition of exon 7 splicing [58]. Modification of splicing through TSL2 requires further study. It can be a good target for screening small molecules [59].

In 2006, Singh et al. discovered an ISS *SMN2* intron-7 in the human *SMN1/2* gene, named ISS-N1 [60]. ISS-N1 is a sequence located immediately downstream of the 5′ss of exon 7. It is 15-nt long and binds positions 10 to 24 of intron 7, producing a strongly inhibitory effect on exon 7 inclusion [61]. Interestingly, ISS-N1 deletion reduced the requirement for positive cis-elements in exon 7 inclusions, an effect similar to the A54G mutation [51]. Blocking ISS-N1 with low concentrations of antisense oligonucleotide (ASO) effectively increased SMN protein levels in studies in mouse models or fibroblasts collected from SMA patients. This demonstrates the high availability and binding efficiency of ISS-N1 for this group of compounds [47]. Importantly, modifying alternative splicing of *SMN2* by targeting ISS-N1 is already used by the ASO drug nusinersen (Spinraza) in the first approved therapy to treat SMA [62].

A more specific target for ASO than ISS-N1 appeared to be the GC-rich sequence (GCRS), which spans from the 7th to 14th position of intron 7 overlapping ISS-N1. Studies on SMA type I patient cells and severe SMA mouse models demonstrated the efficacy of 8-mer ASO binding to GCRS, which not only elevated SMN levels but also increased the levels of Gemin 2 and Gemin 8 factors involved in snRNP biogenesis and Tra2-β1 and hnRNP Q, responsible for proper RNA splicing [63,64].

GCRS participates in the formation of a 5′ strand of a unique RNA structure called the internal stem formed by long-distance interactions (ISTL-1). 279-nts divides the two 8-bp ISTL1 strands. The last position of ISTL-1 and the first position of ISS-N1 is the C residue located at the 10th intronic position (10C) [65]. Interestingly, an experiment was conducted by targeting ISS-N1 with ASOs of equal length (14-mer). F-14 binding to the first nucleotides of ISS-N1, including 10C, promoted exon 7 inclusion, while L-14 targeting the terminal positions of ISS-N1 without 10C had the opposite effect, promoting exon 7 exclusion [66]. ISTL-1 has been shown to negatively regulate exon 7 splicing independently of snRNP A1. Destabilization of ISTL-1 induces abolition of long-distance interaction (LDI) mediated by C10. ASO-mediated sequestration of the 3′ strand of ISTL-1 and upstream sequences form ISS-N2, which results in correction of alternative splicing and restoration of full-length SMN secretion [66,67].

Sequences flanking exon 7 such as Element 1 located in intron 6 also appear to be promising therapeutic targets. Element 1 is a cis-element that is an extended inhibitory sequence located upstream of the 3′ss exon 7 [68]. The effect of promoting full-length SMN expression was demonstrated by targeting Element 1 with morpholino ASOs in mouse models [69].

## 4. Antisense Oligonucleotides

A novel approach to the therapy of SMA and other genetically determined diseases is represented by the use of ASO [70]. ASOs are short (about 15–30 nucleotides in length), single-stranded molecules of chemically modified nucleic acids or nucleotide analogs that, on the basis of complementarity, recognize and bind target sequences in RNA through Watson-Crick base pairing [71,72]. Depending on the binding site, ASOs affect transcript inactivation or splicing, leading to changes in exon content [73]. ASOs are designed to pair bases and form a steric block for binding splicing factors to pre-mRNAs. RNA alters the recognition of splicing sites by the spliceosome, leading to a change in the normal splicing of the target transcript [72]. Modified sequence-dependent ASOs can appropriately lead to the exclusion or inclusion of an exon that would have been excised, as is the case in SMA [72].

This relies on exon 7 appearing in the mature *SMN2* transcript. It is necessary to block the action of the intron folding silencer. Antisense oligonucleotides recognize, on the basis of nitrogenous base complementarity, precisely this *SMN2* pre-mRNA fragment and sterically block its recognition by appropriate proteins. This prevents the formation of a complex that would inhibit detection of the exon/intron boundary. Subsequently, the split between exon 7 and intron 7 is detected, resulting in the incorporation of exon 7 into the mature transcript [74]. The use of appropriate ASOs to treat spinal muscular atrophy allows exon 7 to be incorporated into the transcript of the *SMN2* gene.

**Figure 3:**
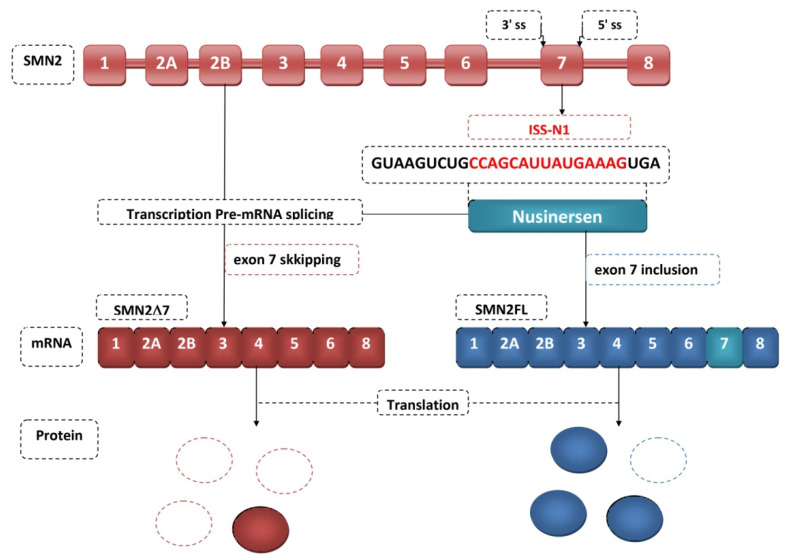
Nusinersen therapeutic mechanism.

The first drug approved for the treatment of SMA was nusinersen (Spinraza TM) [75]. Its discovery took place in 2010 [76]. It is an antisense oligonucleotide that binds to the splicing inhibitory sequence of intron 7. Nusinersen is an 18-mer oligonucleotide in which the sugar-phosphate backbone has been chemically modified [77]. Nusinersen complementary hybridizes to ISS-N1 to block hnRNP recruitment, resulting in the inclusion of exon 7 incorporation into the *SMN2* transcript, resulting in higher levels of fully functional SMN protein [78] (Figure 3). This protein is associated with SMA. As the amount of SMN protein increases, the degeneration of motor neurons stops and the disease progresses [78]. This influences a patient’s longer survival, better motor function and faster achievement of milestones. Nusinersen does not cross the blood-brain barrier and therefore requires intrathecal administration. The half-life of the drug is 163 days, and doses must be repeated throughout life [79]. Monitoring in patients of thrombocyte count, prothrombin time, partial thromboplastin time, and urinalysis results is necessary during nusinersen therapy because it can lead to thrombocytopenia and coagulation disorders, and is nephrotoxic [80]. It can be used to treat all types of SMA [81]. Another breakthrough in the treatment of SMA was the December 2016 approval by the American Food and Drug Administration of the medicine nusinersen (SpinrazaTM), also known as ISIS-SMNRx or ISIS [82]. If a patient does not reach an advanced stage of muscle atrophy, appropriate physiotherapy and multidisciplinary care in combination with nusinersen can produce a significant improvement in the condition of the treated patient [83,84] (Table 1).

## 5. Small Molecules

A project led by PTC-Roche (PTC Therapeutics, South Plainfield, New Jersey and Hoffmann-La Roche, Basel, Switzerland) to identify an orally available molecule to treat SMA began about a decade ago. Both groups identified small molecules and reported three orally delivered compounds, namely SMN-C1 (isocoumarin), SMN-C2 (coumarin), and SMN-C3 (pyridopyrimidinone derivative); each promoted exon 7 inclusion from *SMN2* [92]. Small molecules can exhibit high selectivity, affecting the modulation of RNA folding of only one or a few genes, among the many thousands of genes expressed in cells [93]. Most drugs are inhibitors of enzyme proteins or receptors. It is worth noting that it is possible to obtain modulators of interactions in RNA-RNA and RNA-protein complexes [94]. Risdiplam is being developed by Roche, PTC Therapeutics Inc and the SMA Foundation for the treatment of SMA. In August 2020, the European Medicines Agency (EMA) approved the use of risdiplam to treat patients with the *SMN1* gene mutation [95]. This experimental drug manifests high selectivity for modulation of RNA folding against the *SMN2* transcript. It affects the alternative splicing of a small pool of other genes, such as *FOXM1, MADD* or *STRN3* [94,95]. Risdiplam is not a substrate for the transport protein MDR1, and thus crosses the blood-brain barrier well. It is properly distributed in the CNS and peripheral tissues of mice, rats, and monkeys after single or repeated oral or intraperitoneal administration. Risdiplam also increased levels of functional SMN protein in the CNS and peripheral tissues of mouse models of SMA [96].

Risdiplam is a highly potent *SMN2* splicing modifier that increases exon 7 inclusion in SMN2 mRNA transcripts in in vitro assays and in transgenic mouse models of SMA [95,96,97]. Risdiplam binds to the *SMN2* transcript at two sites—the exonic splicing enhancer 2 (ESE2) in exon 7 and 5′ss of intron 7, thereby dislocating hnRNPG and enhancing 5′ss recognition and binding by U1snRNP. This results in exon 7 not being excised from the transcript and the full SMN protein being able to be synthesized [94,98]. Risdiplam can also increase the binding of far upstream element binding protein 1 (FUBP1) and KH-type splicing regulatory protein (KHSRP) splicing modulators to the *SMN2* pre-mRNA complex, activating *SMN2* splicing [99]. Some of the first preclinical studies have shown that risdiplam can reach the central nervous system and peripheral organs in vivo and can lead to significant increases in SMN protein levels in blood, brain, and muscle, with increased survival in various mouse models of SMA [100,101]. The advantage of this drug is the oral route of administration [102]. Preclinical studies allow for hypothesizing the possibility of a therapeutic effect also in tissues other than the nervous system [102]. This is particularly important because numerous studies in human and animal models indicate that SMA may be considered a multisystem disorder with involvement of the neuromuscular junction, gastrointestinal tract, cardiovascular system, and lung and liver tissues [103,104]. According to the Food and Drug Administration (FDA), on 7 August 2020 risdiplam was approved for the treatment of spinal muscular atrophy in adults and children 2 months of age and older [105]. A recent study analyzing the administration of risdiplam to infants from 1–7 months of age (type 1 SMA) has led to increased expression of functional SMN protein in the blood [100].

Branaplam is another small molecule, administered orally, that modulates *SMN2* splicing with high specificity. It is currently in Phase 2 clinical trials [101,106]. It has been shown to modulate splicing, increase full-length SMN protein levels, and increase survival in a mouse model of severe SMA [107]. The mechanism of action is similar to risdiplam.

To the best of our knowledge, two more molecules PK4C9 and TEC-1, according to recent reports, increase exon 7 SMN2 inclusion with high specificity [59,101,107]. TEC-1 permeabilizes the central nervous system and confers therapeutic efficacy in a mouse model of SMA [59,108]. PCK4C9 targeting the TSL2 tri-loop appeared to cover the “3′-cluster,” a negative element identified by in vivo selection of the entire exon 7 [109,110] (Table 2).

## 6. Future Prospects

**Figure 4:**
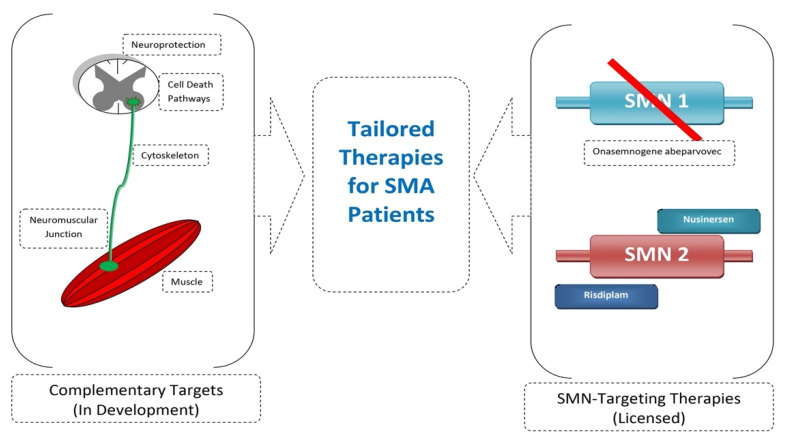
Combined SMN-independent and SMN-dependent therapy as future direction in SMA therapy.

The advancement of SMA therapies has allowed many patients to survive and improve their lives. Current drugs focus on replacing the *SMN1* gene (onasemnogene abeparvovec) or changing *SMN2* splicing (nusinersen, risdiplam). Work is currently underway on a complementary treatment independent of SMN. This applies, for example, to neuroprotective drugs, nerve connection stabilizers, myostatin inhibitors, or activators of muscle function [106,119,120,121] (Figure 4). Many studies also emphasize the importance of early diagnosis and treatment implementation, even presymptomatically. Efforts should be made to develop effective neonatal screening for SMA and to update treatment regimens due to the evolving phenotype of the disease [122,123,124]. Currently, the most important modifier of SMA is the *SMN2* copy number; however, it has been noticed that patients with the same *SMN2* copy number show a difference in the disease phenotype. Work is currently underway to find new biomarkers of disease evolution [125,126,127]. It is also important to provide multidisciplinary care for treated children [128,129].

## 7. Conclusions

The use of the molecular basis of SMA by drugs such as nusinersen, Zolgensma, and risdiplam has brought significant benefits to patients with this fatal disease. Modification of exon 7 alternative splicing turns out to be a key mechanism and target for further research. Current research proves that this therapeutic strategy can effectively increase the level of the SMN protein and, as a result, reduce the course of the disease. Early diagnosis and initiation of treatment in the patient allow for the extension of lifespan and the achievement of milestones. Work is underway on the implementation of other compounds in SMA therapy that bind to factors involved in the regulation of splicing.

## Figures and Tables

**Table 1:** Comparison of potential and the newest targets for ASOs in SMA therapy.

	Aim of the Study	Mechanism of Action	Results	References
1	Therapeutic effect of short ASO on two mouse models of SMA: healthy, adult Smn heterozygous mice containing human *SMN2* and 5058-Hemi SMA mice	Blocking GCRS	Restoring the correct splicing of exon 7 and consequently the production of full-length SMN. Proving efficacy of short ASOs in pathology and expanding the range of ASO-based substances for use in SMA therapy	Keil et al. [64] 2014
2	Variable mechanisms regulating splicing of exon 7 in SMA-patient-derived GM03813 cell line	Targeting ISS-N1 Targeting ISS-N2	Increasing SMN level by stimulating exon 7 inclusion by sequestration of ISS-N1 Increasing SMN and Gemin2 levels with disruption of the 3′ strands of ISTL1 and ISTL2 caused with ISS-N2 blocking Long distance interactions between intron sequences are crucial in understanding the mechanism of disrupted SMA splicing.	Singh et al. [85] 2015
3	Improvement of ASO targeting Element 1 in SMNΔ7 mouse model	Binding potential intronic splicing silencer—E1 in upstream of exon 7 *SMN2*	*SMN2* splicing modification to produce full-length SMN One of the compounds being tested, E1MOv11, has the potential to become a stand-alone ASO in the clinic, but it is critical to develop combination therapy with drugs that act on other SMA pathomechanisms.	Osman et al. [69] 2016
4	Evaluation of the tolerability, safety, pharmacokinetics, and clinical efficacy of nusinersen in cohort of 28 children with type 2 and type 3 SMA aged 2–14 years	Targeting ISS-N1	Initiating exon 7 inclusion resulting in full-length SMN expression No safety issues found with 9 mg nusinersen dose, supporting study of higher dose.	Chiriboga et al. [86] 2016
5	ASO effect targeting deep intronic structures to restore full-length SMN expression in allele C (C/C) mice model	Targeting ISS-N2	A small peripheral increase in SMN alleviates SMA symptoms in a gender-specific manner—restoration of peripheral SMN production has a significant impact on testicular function.Targeting deep intron sequences is effective and has great therapeutic potential, so there is a need for further research into this strategy.	Howell et al. [87] 2017
6	Locked nucleic acid (LNA)-based antisense oligonucleotides (LNA/DNA mixmers) as therapeutic strategy using SMA patient fibroblasts	Targeting ISS-N1	LNA/DNA mixmer-based antisense oligonucleotide may be a potential candidate for SMA therapy.	Touznik et al. [88] 2017
7	Mechanisms influencing ASOs-induced intron retention. ASOs impact on transcript and protein expression in SMA patient fibroblasts	Targeting *SMN2* exon 8 to slowing transcription	Induction of exon/intron 7 retention Model probably not useful for SMA patients. May prove beneficial in diseases in which protein repression is crucial for therapy, e. g., cancers	Flynn et al. [89] 2018
8	Safety and efficacy of nusinersen administration in children with cohort of 126 children with SMA who had symptom onset after 6 months of age	Targeting ISS-N1	Children with later-onset SMA showed a significant improvement in motor function after nusinersen administration compared to control group.	Mercuri et al. [90] 2018
9	Safety and efficacy of nusinersen in the pre-symptomatic period or at the onset of symptoms in cohort of 25 children with genetically diagnosed SMA at a median follow-up of 2. 9 years	Targeting ISS-N1	Early screening and implementation of nusinersen therapy in the presymptomatic period significantly increases the chances for successful therapy and further normal motor development of the child treated for SMA.	De Vivo et al. [91] 2019
10	Effects of nusinersen on the behavior of Cajal bodies (CBs) in SMN∆7 mice	Targeting ISS-N1	Improving motor function and preventing α-motoneuron loss Selective restoring of SMN expression in the spinal cord	Berciano et al. [47] 2020

**Table 2:** Comparison of potential and the newest targets for small molecules in SMA therapy.

	Aim of Study	Mechanism of Action	Results	References
1	Identification and optimization of a pyridazine class of orally bioavailable, small molecules enhancing inclusion SMN exon 7 in mice.	Stabilization of U1 snRNP and *SMN2* pre-mRNA complex Enhancing selectively the binding affinity of U1 snRNP to 5′ss.	Modification of splicing through small sequence-specific molecules can be used in various splicing-related diseases.	Palacino et al. [107] 2015
2	Orally deliverable small molecules correcting alternative splicing of the *SMN2* gene exon 7 in SMA Δ7 mice, SMA patient fibroblasts and rats	Enhancing of the U1−pre-mRNA interaction at the 5′ splice site of *SMN2* intron 7.	Reduction of disease manifestations and a significant increase in the median survival time in models after tested molecules administration Supporting the development of an orally administered small molecule for the treatment of patients with SMA	Woll et al. [111] 2016
3	SMN-C1 in the context of preclinical data for the clinic and further therapeutic development of this series of molecules for the treatment of SMA tested in SMN∆7 mice model.	Increasing the levels of spliceosomal and U7 snRNAs. Correcting RNA processing defects induced by SMN deficiency.	Lower dose SMN-C1 increases long-term survival of SMN∆7 mouse model with partially corrected phenotype. Higher dose of SMN-C1 results in increased body weight, longer survival, and in addition, improved SMN-dependent RNA processing, spinal cord histopathology, and neuromuscular junctions.	Zhao et al. [112] 2016
4	Improvement of coumarin and isocoumarin series, optimization of the pyridopyrimidinone series in C/C-allele SMA mouse model, SMA patient fibroblasts, spinal motor neurons SMA type I and II, and patient-derived induced pluripotent stem cells.	Induction of alternative splicing of *SMN2* to exon 7 inclusion.	Discovery of selective small molecules that modify alternative splicing.	Ratni et al. [113] 2016
5	New advanced chemotype of a small molecule discovered with SMA Δ7 mice model.	Modification of *SMN2* alternative splicing to increase SMN levels.	Discovery of the two orally administrated *SMN2* splicing modifiers.	Pinard et al. [114] 2017
6	Identification of a pyridazine *SMN2* pre-mRNA splicing modulator and optimization to branaplam in SMNΔ7 mouse model and SMA patient fibroblasts.	Stabilization of the interaction between the spliceosome and *SMN2* pre-mRNA.	Branaplam treatment increased full-length SMN RNA and protein levels and extended survival.	Cheung et al. [115] 2018
7	SMN-C2 and SMN-C3 promoting binding FUBP1 and KHSRP to the *SMN2* pre-mRNA complex in 293T cells.	SMN-C2—binding to the AGGAAG *SMN2* pre-mRNA exon 7 SMN-C3—hypothetically targets a sequence of RNA on or close to exon 7 or a splicing regulatory protein or protein complex that is specific to exon 7.	Small molecules complementary to nucleic acids modulate pre-mRNA splicing and can have a therapeutic influence on SMA. Future studies should concern recognition sequence of FUBP1 and KHSRP and their contribution in splicing regulation.	Wang et al. [99] 2018
8	Tolerance and safety testing of RG7800 in clinical trials in cohort of Male subjects aged 23–45 years, thirteen patients with SMA, aged 13–53 years.	Modification of splicing toward promoting full-length SMN expression and downregulating SMNΔ7.	RG7800 is safe and well tolerated, and that the level of SMN after oral administration increases by twofold over the baseline concentration which may be associated with future therapeutic benefits.	Kletzl et al. [116] 2019
9	Safety, tolerability, pharmacokinetics, and pharmacodynamics of risdiplam in cohort of 25 adult males, aged 18–45 years. Itraconazole effect on the pharmacokinetics of risdiplam.	Highly specific for pre-mRNA *SMN2* splicing modifier	The tested doses of risdiplam were well tolerated and safe, and produced the desired effect of increasing full-length *SMN2* pre-mRNA levels CYP3A inhibitors in the form of itraconazole have little effect on the pharmacokinetics of risdiplam.	Sturm et al. [117] 2019
10	Preclinical characterization and prospects of TEC-1 using SMAΔ7 mice and SMA patient fibroblasts.	Binding to purine-rich regions within exon 7 Interaction with the major groove of the RNA duplex generated by the 5ʹ splicing site of exon 7 and U1 snRNA17	Low risk of acute or chronic side effects Promising for the long-term treatment of patients with SMA Potentially higher therapeutic window compared to the SMN-C series.	Ando et al. [2] 2020
11	Drugs that boost the minigene reporter signal within the context of Drosophila motor neurons	Promoting the inclusion of *SMN2* exon 7 in a dose-dependent manner	Increasing SMN and SRSF1 levels and decreasing level of hnRNP1 with moxifloxacin The effects of moxifloxacin need to be tested in murine models as a potential SMA therapy or scaffold for other variant molecules.	Konieczny and Artero [118] 2020

## Data Availability

Not applicable.
